# Control of somatic membrane potential in nociceptive neurons and its implications for peripheral nociceptive transmission

**DOI:** 10.1016/j.pain.2014.08.025

**Published:** 2014-11

**Authors:** Xiaona Du, Han Hao, Sylvain Gigout, Dongyang Huang, Yuehui Yang, Li Li, Caixue Wang, Danielle Sundt, David B. Jaffe, Hailin Zhang, Nikita Gamper

**Affiliations:** a Department of Pharmacology, Hebei Medical University, Shijiazhuang, PR China; b Faculty of Biological Sciences, School of Biomedical Sciences, University of Leeds, Leeds, UK; c Department of Biology, University of Texas at San Antonio, San Antonio, TX, USA

**Keywords:** DRG, Nociceptor, M channel, KCNQ, K2P, Ion channel, Pain

## Abstract

Peripheral sensory ganglia contain somata of afferent fibres conveying somatosensory inputs to the central nervous system. Growing evidence suggests that the somatic/perisomatic region of sensory neurons can influence peripheral sensory transmission. Control of resting membrane potential (E_rest_) is an important mechanism regulating excitability, but surprisingly little is known about how E_rest_ is regulated in sensory neuron somata or how changes in somatic/perisomatic E_rest_ affect peripheral sensory transmission. We first evaluated the influence of several major ion channels on E_rest_ in cultured small-diameter, mostly capsaicin-sensitive (presumed nociceptive) dorsal root ganglion (DRG) neurons. The strongest and most prevalent effect on E_rest_ was achieved by modulating M channels, K2P and 4-aminopiridine-sensitive K_V_ channels, while hyperpolarization-activated cyclic nucleotide-gated, voltage-gated Na^+^, and T-type Ca^2+^ channels to a lesser extent also contributed to E_rest_. Second, we investigated how varying somatic/perisomatic membrane potential, by manipulating ion channels of sensory neurons within the DRG, affected peripheral nociceptive transmission in vivo. Acute focal application of M or K_ATP_ channel enhancers or a hyperpolarization-activated cyclic nucleotide-gated channel blocker to L5 DRG in vivo significantly alleviated pain induced by hind paw injection of bradykinin. Finally, we show with computational modelling how somatic/perisomatic hyperpolarization, in concert with the low-pass filtering properties of the t-junction within the DRG, can interfere with action potential propagation. Our study deciphers a complement of ion channels that sets the somatic E_rest_ of nociceptive neurons and provides strong evidence for a robust filtering role of the somatic and perisomatic compartments of peripheral nociceptive neuron.

## Summary

We identified major ion channels influencing the resting membrane potential of nociceptive sensory neurons and demonstrated that changes of somatic/perisomatic membrane potential of these neurons can strongly influence peripheral nociceptive transmission.

## 1 Introduction

In contrast to the majority of central nervous system neurons, peripheral somatosensory neurons normally generate action potentials (APs) at peripheral nerve endings, not at the axon hillock [2], [3]. While somatic APs and electrogenesis are not required for AP propagation from the periphery to the spinal cord [4], sensory neuron somata are electrically excitable [3], [12], [111], [122], and ectopic somatic activity [3], [12], [77], [111], [122], along with ectopic peripheral fibre activity [22], [23], [125], is thought to contribute to many chronic pain conditions. Moreover, measurements [28], [33], [110], [113] and simulations [79] suggest that the axonal bifurcation (t-junction) within dorsal root ganglia (DRG) influences the transmission of spikes on their way to the spinal cord. Hitherto unexplained recent clinical studies have established that direct electrical stimulation (“neuromodulation”) of the DRG provides efficacious pain relief in neuropathic pain patients [20], [95]. Taken together, these findings suggest that sensory ganglia may play a much stronger role in peripheral nociceptive transmission than is generally accepted. Moreover, sensory ganglia may represent a novel target for pain therapeutics [95]. Yet, surprisingly little has been done so far to directly test how electrophysiological properties of somatic/perisomatic compartment of sensory neuron affect peripheral somatosensory transmission.

The aims of this study were 1) to identify major ion channels influencing the resting membrane potential (E_rest_) of nociceptive DRG neurons and 2) to investigate if (and how) manipulation with the activity of these channels within the somatic/perisomatic compartments of DRG would affect peripheral nociceptive transmission. In the first part we focused on the ion channels that are known to be expressed in nociceptive DRG neurons and would be expected to be active at, and possibly contribute to, the E_rest_ of these neurons. These channels included 4-aminopiridine (4-AP)-sensitive voltage-gated K^+^ (K_V_) channels [26], slow-activating M channels (Kv7, KCNQ) [26], [58], [71], [74], [92], [93], [100], [101], 2-pore K^+^ “leak” channels (K2P) [1], [80], [115], sodium-activated K^+^ channels (Slo2.x, K_Na_) [32], [91], [116]; hyperpolarization-activated cyclic nucleotide-gated channels (HCN) [13], [30], [31], [128], low voltage-activated T-type Ca^2+^ channels (Ca_v_3.x) [50], [90], [107], [114], and voltage-gated Na^+^ channels (VGNC) [6], [60]. These analyses identified M channels, 4-AP-sensitive K_V_ and K2P “leak” channels as those having most significant influence over the E_rest_. We then investigated how the hyperpolarization of somatic/perisomatic E_rest_ in the DRG achieved by modulating somatic/perisomatic ion channel activities can influence nociceptive transmission in vivo. Finally, a computational model of a nociceptive DRG neuron was used to better understand how the relationship between morphology, membrane potential, and ion channels active at E_rest_ influence nociceptive signal propagation. Our study identifies major ion channels that set somatic E_rest_ of nociceptive neurons and provides firm evidence for a much stronger role of sensory ganglia in the peripheral nociceptive transmission than is generally thought.

## 2 Materials and methods

### 2.1 Neuronal cultures and slice preparation

DRG neurons were cultured as described previously ([74], [76]; see [59] for step-by-step protocol). Briefly, adult male Sprague-Dawley rats (180-200 g) were humanely euthanized by cervical dislocation under the isoflurane anaesthesia. DRG from all spinal levels were removed and treated at 37°C in Hank’s Balanced Salt Solution supplemented with collagenase (1 mg/mL; Sigma-Aldrich, St. Louis, MO, USA) and dispase (10 mg/mL; Invitrogen, Life Technologies, Grand Island, NY, USA) for ∼30 minutes. Ganglia were then gently triturated, washed twice, and resuspended in 600 μL culturing media (approx. 500,000 cells per isolation); this suspension was then plated as dense cultures onto glass coverslips coated with poly-D-lysine and laminin. Neurons were cultured for 2 to 5 days. No nerve growth factor was added to the culture to avoid inflammatory insult; we found that densely plated cultures survive well without trophic factors added. It is of note that our dissociation protocol provides cultures that are enriched with small-diameter, high-threshold (presumed nociceptor) neurons because large-diameter, low-threshold mechanoreceptors mainly die during trituration due to the mechanical overstimulation, unless specifically protected [36], [59].

For sharp electrode recording, DRG slices were prepared from 12-day-old Wistar rats as described earlier [101], with slight modifications. Briefly, DRG were embedded in agar and sliced (300 μm) in ice-cold extracellular solution using a vibrating blade microtome (VT100S; Leica Microsystems, Buffalo Grove, IL, USA). Slices were then stored at room temperature for the remainder of the day in carbogenated (95% O_2_-5% CO_2_) extracellular solution containing (in mM): 115 NaCl, 25 NaHCO_3_, 11 D-Glucose, 5.6 KCl, 2 MgCl*_2_*, 1 NaH_2_PO_4_, and 2.2 CaCl_2_ (pH 7.4).

### 2.2 Electrophysiology

Whole-cell and perforated patch recordings in current clamp configuration were performed at room temperature (unless indicated otherwise). Patch pipettes (resistance 2–4 MΩ) were fabricated from borosilicate glass capillaries using a DMZ-universal horizontal puller (Zeitz, Martinsried, Germany) or a Sutter P-97 puller (Sutter, Novato, CA, USA). Currents were amplified and recorded using an EPC-10 patch amplifier and Patchmaster 2.2 software (HEKA Electronik, Lambrecht, Germany) or an Axon patch 700B amplifier and pCLAMP 10.0 software (Axon Instruments, Union City, CA, USA), and were sampled at a frequency of 5 kHz. Liquid junction potentials were calculated with the algorithm developed by P.H. Burry [7] using pCLAMP software and subtracted post acquisition. Continuous current-clamp recording with no current injection was used for E_m_ monitoring. Linear ramps of currents from 0 to 1 nA (1-second duration) were injected for measuring rheobase and other AP parameters. The extracellular solution contained (in mM): 160 NaCl, 2.5 KCl, 5 CaCl_2_, 1 MgCl_2_, 10 HEPES, and 8 glucose, pH 7.4. The intracellular solution for perforated patch experiments [70] contained (in mM): 150 KCl, 5 MgCl_2_, 10 HEPES, pH 7.4. (with 0.2-0.4 mg/mL amphotericin B, Sigma). The intracellular solution for whole-cell recordings from cultured DRG neurons contained (in mM): 150 KCl, 5 MgCl_2_, 10 HEPES, 4 adenosine triphosphate (ATP; magnesium salt), pH 7.4. For whole-cell recordings from DRG slices, extracellular solution contained (in mM): 115 NaCl, 25 NaHCO_3_, 5.6 KCl, 1 NaH_2_PO_4_, 1 MgCl_2_, 2.2 CaCl_2_, 11 glucose, pH 7.4, and intracellular solution contained (in mM) 130 KCl, 5 MgCl_2_, 4.63 CaCl_2_, 5 EGTA, 5 HEPES, 3 ATP (dipotassium salt), pH 7.4. Whole-cell current clamp recordings were performed as previously described [101].

Sharp electrode recordings were performed from DRG slices held in a submerged-type chamber and perfused with carbogenated extracellular solution (4-5 mL/min) at room temperature. Electrodes were pulled using a DMZ-universal horizontal puller to resistances of 70-120 MΩ when filled with a solution containing 1 M K-acetate (plus 1 mM KCl; pH 7.2 adjusted with acetic acid). Some recordings were performed with electrodes filled with 1 M KCl (plus 10 mM HEPES titrated to 7.2 with potassium hydroxide). Recordings were made using an SEC-05L amplifier (npi electrotonic, Tamm, Germany) and digitized (10 kHz) with a PC-based system (Digidata 1200 and Clampex 9.3, Molecular Devices, Sunnyvale, CA, USA) and analysed off-line (Clampfit 10.1). To measure the rheobase and to analyse AP properties, a family of 600-ms current injections (between −0.35 and +1 nA with 0.05 nA increment) was used. Because liquid junction potential should be <1 mV [89], no correction was applied.

To identify neurons as nociceptive, capsaicin (1 μM) has been applied at the end of the recording in all recording paradigms. Due to the small number of capsaicin-insensitive neurons and due to the fact that it was not always possible to apply capsaicin (eg, due to the premature loss of the recording), data from capsaicin-sensitive and capsaicin-insensitive neurons were not analysed separately.

### 2.3 Experiments with recombinant channels

In experiments testing specificity of K_Na_-modulating drugs, plasmids encoding human Kv7.2 and Kv7.3 (GenBank accession no. NM000218 and AF091247) were transfected into Chinese hamster ovary (CHO) cells using Lipofectamine 2000 (Invitrogen). In experiments testing effect of ST101 on Cav3.2, the plasmid encoding human Cav3.2 (GenBank accession no. AF051946; kind gift from Prof. Chris Peers, University of Leeds, UK) was transfected into human embryonic kidney (HEK293) cells and whole-cell recordings were performed. The recordings were made using an Axon 700B patch-clamp amplifier (Axon Instruments); signals were filtered at 2 kHz and analysed using pCLAMP 10 (Axon Instruments) and Origin 7.5 (OriginLab Corporation, Northampton, MA, USA).

### 2.4 Atomic absorption spectroscopy Rb^+^ efflux assay

Rb^+^ efflux assay to study the modulation of M channels has been described in detail previously [97]. Briefly, CHO cells stably transfected with Kv7.2 and Kv7.3 were grown to confluence in 96-well plates. For Rb^+^ loading, the cell culture medium was gently removed, the monolayer was washed once with 200 μL of Rb^+^ loading buffer containing (in mM): 5.4 RbCl, 5 glucose, 25 HEPES, 150 NaCl, 1 mM MgCl_2_, 0.8 NaH_2_PO_4_, and 2 CaCl_2_ (pH adjusted to 7.4 with NaOH). Cells were loaded in the same buffer (200 μL per well) for 3 hours at 37°C, 5% CO_2_. After loading, cells were washed gently 3 times with wash buffer containing (in mM) 5.4 KCl, 25 HEPES, 150 NaCl, 1 MgCl_2_, 0.8 NaH_2_PO_4_, and 2 CaCl_2_ (pH adjusted to 7.4 with NaOH). The wash buffer was then replaced with 200 μL of depolarization buffer containing (in mM) 20 KCl, 25 HEPES, 130 NaCl, 1 MgCl_2_, 0.8 NaH_2_PO_4_, and 2 CaCl_2_ (pH adjusted to 7.4 with NaOH). The ion channel modulators were added to depolarization buffer. Channel activation was maintained for 10 minutes. Supernatant (200 μL from each well) was collected and transferred to a new 96-well plate before measurement. The concentration of Rb^+^ in the cell supernatants was determined using an automated Ion Channel Reader 8000 flame atomic absorption spectrometer (Aurora Biomed, Vancouver, BC, Canada). The concentration-response curves were fit with the equation y = A_2_ + (A_1_ − A_2_)/1 + (x/x_0_)^p^), where y is the response; A_1_ and A_2_ are the maximum and minimum response, respectively; x is the drug concentration, and p is the Hill coefficient.

### 2.5 Acute focal application of ion channel modulators to DRG in vivo

All surgical procedures were performed under deep anaesthesia with an intraperitoneal injection of pentobarbital sodium (10-20 mg/kg) in accordance with the Animal Care and Ethical Committee of Hebei Medical University (Shijiazhuang, China) under the International Association for the Study of Pain guidelines for animal use. Focal application of compounds to the DRG in vivo was performed as described before [96], with modifications. Briefly, a midline incision was made at the L4-L6 spinal level of an adult male rat (Sprague-Dawley; 180-200 g), and the L5 was identified at the midpoint of a link between both sides of iliac crest. A 0.8-mm hole (approximately 2 mm off the inferior edge of the transverse process) was drilled through the transverse process over the L5 DRG. Approaching of ganglion was verified by the twitch of the hind paw, at which point the drilling was stopped immediately. A hooked stainless steel blunt-tip cannula (inner diameter 0.64 mm, length 4 mm) was forced into the hole and connected to a polypropylene tube (inner diameter 0.41 mm, length 4.5 mm). The incision was closed with sutures and the cannula was firmly fixed in place with dental cement. Intramuscular injection of benzylpenicillin (19 mg/0.1 mL) was given immediately after surgery. Postoperatively, rats were housed individually in plastic cages with sawdust flooring and supplied with water and food ad libitum. Animals were left to recover for at least 24 hours before the experiments were carried out. Animals developing signs of distress were humanely euthanized by cervical dislocation under the isoflurane anaesthesia.

To evaluate the effect of focal application of ion channel modulators to DRG on the nociceptive processing, 5 μL of retigabine, pinacidil, or ZD7288 solution (each at 200 μM) or saline/vehicle control were injected via the DRG cannula immediately prior to the hind paw plantar injection of 50 μL of bradykinin (200 μM). The animal was returned to the cage and video-recorded for 30 minutes. Time spent licking, flinching, and biting the injected paw over the period of 30 minutes was analysed by the operator blind to the composition of the injected solution.

In order to verify that drug exposure was limited to the DRG, a fluorescent dye, 5(6)-Carboxyfluorescein diacetate *N*-succinimidyl ester (Sigma; 20 μM in 5 μL), was injected via the cannula implanted as described above. Dye injection was performed on animals that received no other injections before; approximately 30 minutes after injection, the animal was sacrificed, both the L5 DRG and proximal inferior part of the lumbar spinal cord were excised, submerged in Tissue-Tek O.C.T. (Sakura, Alphen aan den Rijn, The Netherlands), frozen, and sectioned (15 μm) using a freezing microtome (CM1950, Leica Microsystems). Slices were then analysed for the presence of dye using confocal microscopy (TCS SP5 II, Leica Microsystems).

### 2.6 Computer modelling

A computational model of small-diameter nonmyelinated DRG neuron was constructed and simulated using NEURON (http://www.neuron.yale.edu) [42], [43] on an Intel-based Macintosh computer (Apple Inc, Cupertino, CA, USA). Simulations were analysed using IgorPro analysis software (Wavemetrics, Lake Oswego, OR, USA). Our model neuron had a morphology based on available literature: the soma was 25 μm in diameter [38], [129], with a capacitance of 20 pF, while the diameters of the peripheral and central axons were 0.8 and 0.4 μm, respectively [35], [45], [79], [112]. The stem axon arising from the peripheral axon had a diameter of 1.4 μm and was 75 μm in length, except where noted. Axonal compartments within the DRG were subdivided into 100 sections for computational accuracy [104]. For all compartments, E_rest_ = −60 mV, R_m_ = 10,000 Ωcm^2^, C_m_ = 1 μF/cm^2^, and R_a_ = 100 Ωcm [79]. These parameters resulted in a model with a somatic input resistance of 274 MΩ [38], [129] and an apparent cell capacitance (ratio of membrane time constant and input resistance: τ_m_/R_N_) of 29.6 pF. VGNC and delayed rectifier K^+^ channels were expressed in all compartments with a density of 0.04 S/cm^2^, except at the soma, where VGNC was 0.02 S/cm^2^ [84]. The voltage-dependence of the VGNC was adjusted to be approximately mid-way between values reported for Na_V_1.7 and Na_V_1.8 channels in DRG [18], [106]. M and HCN channels were inserted in the soma, stem axon, and in most simulations, extended 100 μm into the peripheral and central axons. Conductance densities for these channels are reported in units of pA/pF at potentials of −30 mV (M channels V_1/2_) and −100 mV (HCN channels 100% activation). E_leak_ in all compartments was calculated from resting Na^+^, K^+^, and M channel or HCN currents to achieve a E_rest_ of −60 mV [33], [38]. APs were initiated in the peripheral axon distal to the t-junction by depolarizing current steps (0.2 nA, 1-ms duration). Where noted, constant current was injected into the soma (1-100 pA).

### 2.7 Compounds

List of all ion channel modulators used in this study, as well as their abbreviations and concentrations and sources are listed in Table 1. All compounds, except of XE991 (XE), were used at concentrations sufficient to produce maximal effect. XE was used at 3 μM (near IC_80_), as at saturating concentrations it may affect other channels such as eag1 and Kv4.3 [123].

**Table 1:** Ion channel modulators used in this study.

Compound	Acronym	Channel	Mode of action	[X]^a^ μM	Potency	Source
Retigabine	RTG	Kv7	Activator	10	IC_50_ = 1.75 μM	Present study
XE991	XE	Kv7	Inhibitor	3	IC_50_ ∼1 μM for Kv7.2/Kv7.3	[123]
ZD7288	ZD	I_h_/HCN	Inhibitor	10^b^	IC_50_ ∼1 μM (rat DRG)	[82]
Bithionol	BITH	K_Na/_Kv7	Activator	10	K_V_7: EC_50_ = 2.15 μM K_Na_: EC_50_ = 0.77 μM	Present study [127]
Loxapine	LOX	K_Na_	Activator	10	EC_50_ ∼4 μM (Slo2.2)	[9]
Bepridil	BEP	K_Na_	Inhibitor	20	IC_50_ ∼1 μM (Slo2.2)	[127]
4-Aminopiridine	4-AP	Kv	Inhibitor	2000^c^	IC_50_ ∼200 μM (Kv1.x)	Tocris
Pinacidil	PIN	K_ATP_	Activator	10	EC_50_ ∼1.5 μM	[34]
Glibenclamide	GLIB	K_ATP_	Inhibitor	10	IC_50_ ∼10 nM (rat DRG)	[55]
Tetrodotoxin	TTX	Na_v_	Inhibitor	0.1	1-10 nM	Tocris
A803467	A803	Na_v_1.8 Na_v_1.9	Inhibitor	10	EC_50_ = 8 nM (hNa_v_1.8) EC50 ∼1 μM (TTX-r VGNC current in DRG)	[49][117]
Mibefradil	MIB	Ca_v_3	Inhibitor	3	IC_50_ ∼0.15-1.5 μM	[81]
ST101	ST101	Ca_v_3	Activator	0.001	hCa_v_3.2: IC_50_ = 0.12 nM	Present study
Hydroxy-α-sanshool	SAN	K2P	Inhibitor	100	IC_50_ ∼30-50 μM (TASK-1, TRESK)	[8]
Bupivacaine	BUP	K2P	Inhibitor	100	IC_50_ ∼40 μM (TASK-1, TASK-3)	[83]
Lamotrigine	LAM	K2P	Inhibitor	100	IC_50_ ∼30 μM (TRESK in rat DRG)	[54]
Doxapram	DOX	K2P	Inhibitor	100	IC_50_ = 0.4 μM (TASK-1) IC_50_ = 37 μM (TASK-3)	[83]
Riluzole	RIL	K2P Na_v_	Activator Inhibitor	100	EC_50_ ∼50 μM (TRAAK, TREK-1)	[29]
Capsaicin	CAP	TRPV1	Activator	1	EC_50_ ∼10 nM	[16]

a Stated are the concentrations used in the resting membrane potential experiments; in some other types of experiments different concentrations were sometimes used; these instances are indicated in the text.

b 50 μM was used in sharp electrode recordings from dorsal root ganglion (DRG) slices.

c 1 mM was used in sharp electrode recordings from DRG slices.

### 2.8 Statistics

All data are given as mean ± SEM. In the experiments where normal distribution of data cannot be expected, the following approach has been applied. 1) Differences between groups of paired values were analysed using paired Wilcoxon test. 2) Kruskal-Wallis analysis of variance (ANOVA) was used to compare among multiple groups. 3) Pairs within multiple groups were analysed by Mann-Whitney test with Bonferroni correction. 4) Differences in proportions of responsive cells were analysed using Fisher’s exact test. In the case where normal distribution was confirmed, *t*-test (paired or unpaired, as appropriate) was used; where indicated, multiple groups were compared using one-way ANOVA with Bonferroni post hoc test. Differences were considered significant at *P* ⩽ 0.05. Statistical analyses were performed using Origin 9.0 (OriginLab Corporation, Northampton, CA, USA), Minitab 16 (Minitab Inc, State College, PA, USA), and Prism 6.01 (GraphPad Software Inc, La Jolla, CA, USA).

## 3 Results

Membrane potential (E_m_) of a neuron during resting state (resting membrane potential, E_rest_) results from the steady-state interaction of a number of membrane conductances, mostly represented by ion channels [41], [51], with small contribution by electrogenic pumps [61], [63]. In order to identify ionic conductances that contribute to the somatic E_rest_ of nociceptive neurons, we measured changes in E_m_ (ΔE_m_) in response to pharmacological inhibition or activation of ion channels expected to be active at voltages near the E_rest_. Our main experimental model was cultured small-diameter (∼20 μm) DRG neurons with whole-cell capacitance of 26.3 ± 1.3 pF (n = 32); these neurons were predominantly capsaicin sensitive (71% or 174/245 of such cells responded to 1 μM capsaicin). Responsiveness to capsaicin indicates expression of nociceptive neuron marker TRPV1 [65], and thus, we describe the population of neurons under investigation as predominantly small-diameter nociceptors, although contribution of a small number of neurons of other modalities to this population cannot be excluded.

Accurate measurement of E_rest_ can be influenced by the recording configuration, as well as the experimental preparation. Therefore, we used a combination of approaches to measure E_rest_ of small DRG neurons. Specifically, 1) perforated-patch and 2) whole-cell recordings were made from cultured sensory neurons; 3) sharp electrode recordings and 4) a limited number of whole-cell recordings were made from sensory neurons in acute DRG slices. Remarkably, similar values for E_rest_, near −60 mV, were observed across all experiments (Table 2). For whole-cell experiments there was no significant drift of E_m_ value after the breaking into the neuron, indicating little influence of the intracellular solution exchange during the recording period. Likewise, in sharp-electrode recording experiments, replacing the K-acetate-based pipette solution with the KCl-based solution resulted in no significant difference. Our E_rest_ values were very close to those reported previously [3], [5], [38], [71], [72], [103], [129] and show that E_rest_ of both cultured and acute DRG neuron somata are maintained within the same voltage range.

**Table 2:** Electrophysiological properties of the DRG neuron somata.

Recording mode	E_rest_, mV	AP amplitude, mV	Rheobase, nA
Perforated patch current clamp (culture)	−60.1 ± 0.9 n = 160	62.4 ± 3.0 n = 38	0.24 ± 0.03 n = 38
Whole-cell current clamp (culture); first sweep	−59.3 ± 3.1 n = 9	N/A	N/A
Whole-cell current clamp (culture); steady-state	−60.6 ± 2.0 n = 9	N/A	N/A
Sharp electrode recording (slice); KCl-based pipette solution^a^	−61.9 ± 3.2 n = 10	70.3 ± 5.2 n = 4	0.46 ± 0.01 n = 4
Sharp electrode recording (slice); K-acetate based pipette solution^a^	−60.2 ± 1.2 n = 66	60.0 ± 4.1 n = 20	0.46 ± 0.04 n = 20
Whole-cell current clamp (slice); steady-state	−60.4 ± 3.9 n = 6	N/A	N/A

a All the E_rest_ data except of these recorded with the sharp-electrode technique are corrected for the liquid junction potential. It is assumed that in sharp electrode recording, the junction potential is small (within 1 mV) [89].

### 3.1 Channels contributing to E_rest_ in cultured nociceptive DRG neuron somata

**Fig. 1:**
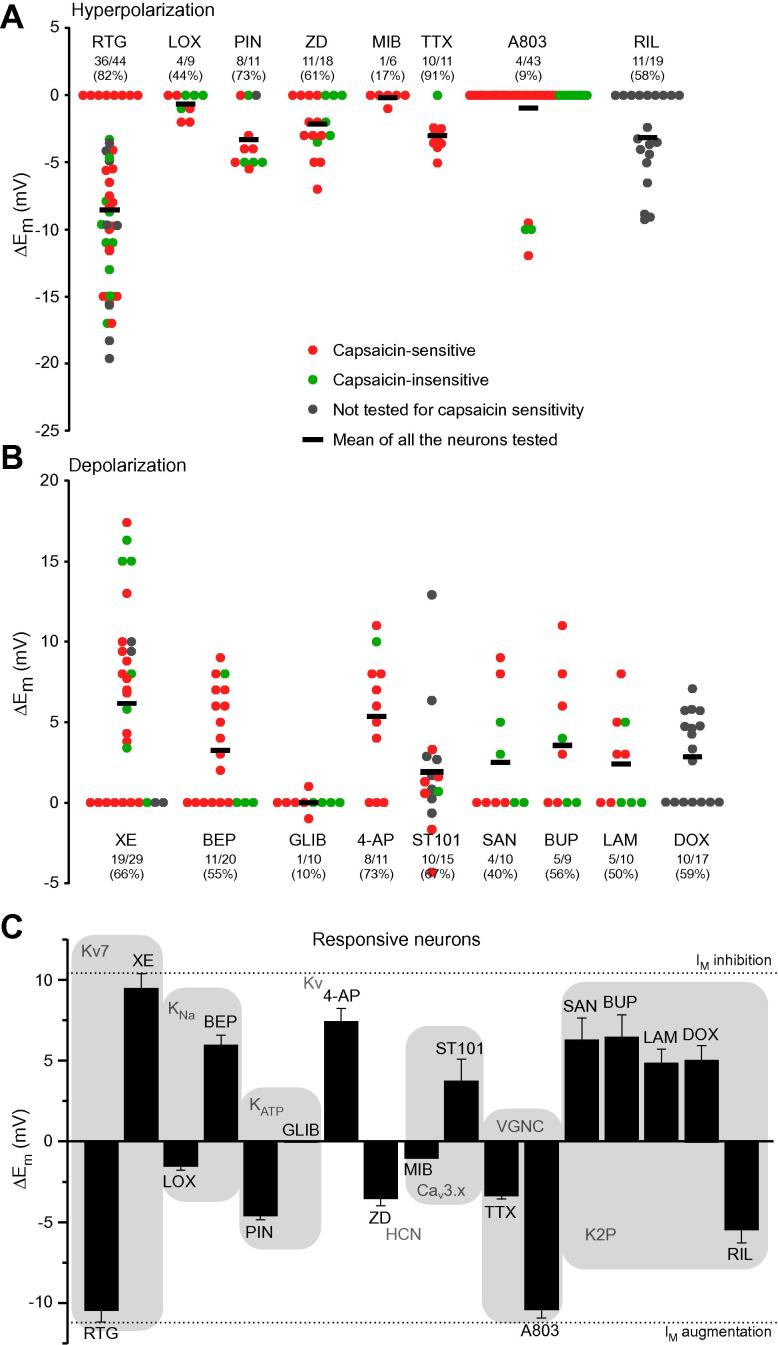
Effects of ion channel modulators on the resting membrane potential of small dorsal root ganglia (DRG) neurons. (A,B) Scatter plots of changes of membrane potential (ΔE_m_) in cultured small-diameter DRG neurons in response to the application of ion channel modulators; panel (A) summarizes hyperpolarizing effects and panel (B) summarizes depolarizing effects (see Table 1 for complete list of compounds). Values from capsaicin-sensitive (red) and -insensitive (green) neurons, as well as from neurons untested for capsaicin sensitivity (dark grey), are identified. Horizontal black bars in every group depict mean values of all neurons tested. Number of responsive neurons out of total neurons tested for each compound are indicated above each group as X/Y, where X is a number of responsive neurons and Y is a total number of neurons tested; percentage of responsive neurons for each compound is also indicated. (C) Bar chart summarizes effects of each of the compounds tested taking into account responsive neurons only (ΔE_m_ changes below 1 mV, an average noise amplitude, were considered as no effect). Dotted lines indicate level of depolarization (upper line) or hyperpolarization (lower line) upon M channel inhibition or enhancement, respectively (as indicated).

**Fig. 2:**
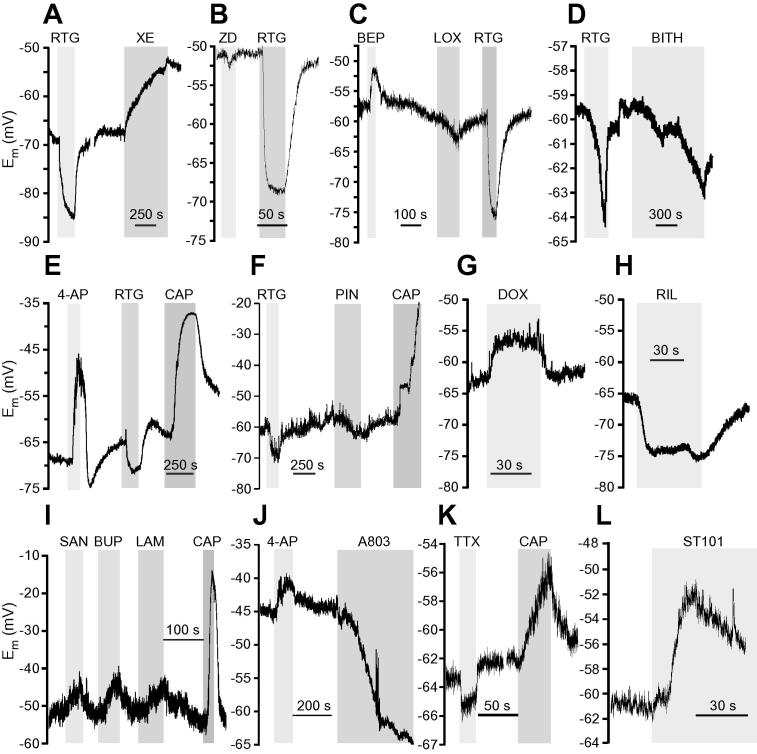
Exemplary recordings of the membrane potential changes in response to modulation of resting currents in small dorsal root ganglia (DRG) neurons. (A–L) Exemplary time courses of E_m_ recorded with whole-cell current clamp from cultured small DRG neurons. The compounds were applied during periods indicated by grey bars (see Table 1 for complete list of compounds). Statistical analysis of responses is presented in Fig. 1 and Table 3.

To identify ion channels contributing to the E_rest_ of nociceptive neurons, we used perforated patch current clamp recordings to measure ΔE_m_ in cultured, small-diameter DRG neurons in response to a set of well-characterized ion channel blockers or enhancers for M channels, K_V_, K_ATP_, K_Na_, HCN, Ca_v_3x, and Na_V_ channels. It has to be noted that if a given ion channel is not tonically active at E_rest_, then its pharmacological activation or enhancement will not inform about the channel contribution to E_rest_. Responses of varying amplitudes were observed for most compounds in various proportions of neurons (Fig. 1). Fig. 1A and B summarizes these experiments, with panel A depicting responses to compounds that produced hyperpolarization and panel B depicting these produced depolarization. Number and percentage of neurons responsive to each compound (those where the absolute value of ΔE_m_ was >1 mV) is listed above each dataset. Fig. 1C compares the mean ΔE_m_ values for each compound determined from the subset of responsive neurons only. Exemplary E_m_ responses are given in Fig. 2 and statistical analysis of the data is summarized in Table 3.

**Table 3:** Effects of ion channel modulators on the resting membrane potential of small DRG neurons; statistical analysis.

	Modulator	Difference from baseline (paired Wilcoxon signed ranks test)	Difference in efficacy (as compared to Kv7 modulator; Kruskal-Wallis ANOVA with subsequent Mann-Whitney test & Bonferroni correction)	Difference in proportion of responding neurons (as compared to Kv7 modulator; Fisher’s exact test). *P*-value
Hyperpolarization				
	RTG	^∗∗∗^	–	–
	LOX	ns	Yes	0.03
	PIN	^∗^	Yes	0.67
	ZD	^∗∗∗^	Yes	0.06
	MIB	ns	Yes	0.001
	TTX	^∗∗∗^	Yes	0.67
	A803	ns	Yes	0.000
	RIL	^∗∗∗^	Yes	0.06

Depolarization				
	XE	^∗∗∗^	–	–
	BEP	^∗^	No	0.55
	4-AP	^∗∗^	No	1.00
	GLIB	ns	Yes	0.000
	ST101	^∗∗^	No	1.00
	SAN	ns	No	0.26
	BUP	^∗^	No	0.70
	LAM	^∗^	No	0.46
	DOX	^∗∗^	No	0.75

DRG, dorsal root ganglia; ANOVA, analysis of variance; RTG, retigabine; LOX, loxapine; PIN, pinacidil; ZD, ZD7288; MIB, mibefradil; TTX, tetrodotoxin; A803, A803467; RIL, riluzole; XE, XE991; BEP, bepridil; 4-AP, 4-Aminopiridine; GLIB, glibenclamide; SAN, hydroxy-α-sanshool; BUP, bupivacaine; LAM, lamotrigine; DOX, doxapram.

^∗^, ^∗∗^, ^∗∗∗^ Denote difference from baseline at *P* ⩽ 0.05, *P* ⩽ 0.01 and *P* ⩽ 0.001 respectively.

The most robust and most abundant responses were to the modulators of M-type K^+^ channels. M channel subunits Kv7.2, Kv7.3, and Kv7.5 are expressed in most DRG neurons [58], [93], [100] and, in particular, in small-diameter, TRPV1-positive nociceptors [76], [100]. M channel enhancer, retigabine (RTG; 10 μM; Figs. 1 and 2A–D) hyperpolarized E_rest_ by −10.4 ± 0.8 mV in 36 responsive neurons (out of a total of 44; 82%), while M channel inhibitor XE (3 μM) induced similar magnitude of depolarization (9.4 ± 0.9 mV) in 19 responsive neurons (out of a total of 29 neurons; 66%). These findings show that M channels significantly contribute to the E_rest_ of an average small-diameter nociceptive DRG neuron soma.

Small-diameter nociceptors express other Kv channels, such as 4-AP-sensitive Kv1.4 [99], [121], Kv2s [10], [119], and Kv3.4 [15], [57] (reviewed in [26]). 4-AP (2 mM), which has little effect on M channels, produced a sizable depolarization of 7.4 ± 0.8 mV in 8/11 neurons (73%; Figs. 1 and 2I, J). This suggests that some K_V_ channels expressed at nociceptor neuron cell bodies are partially open near the E_rest_. The effect of 4-AP was not significantly different from that of XE (Table 3).

**Fig. 3:**
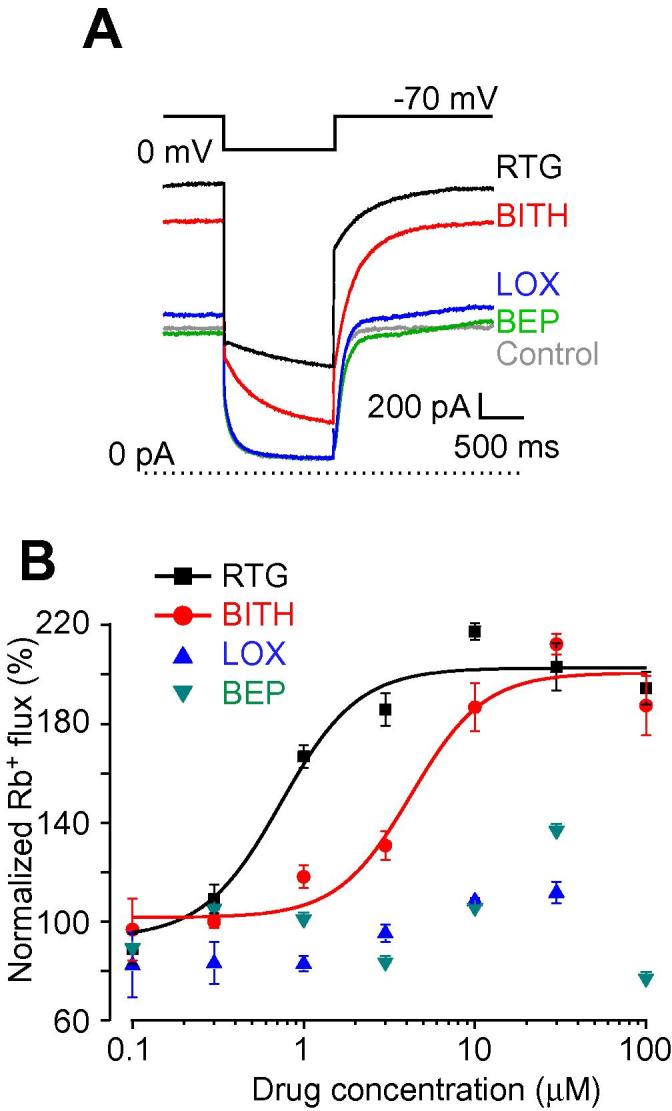
Characterization of K_Na_ channel modulators. (A,B) Bithionol is an M channel enhancer. (A) Effects of BITH (10 μM), RTG (10 μM), LOX (10 μM), and BEP (10 μM) on the recombinant Kv7.2/Kv7.3 channels heterologously overexpressed in Chinese hamster ovary cells. Recordings were made using whole-cell patch clamp; voltage protocol is depicted above the traces. Dotted line represents 0 pA current level. (B) Concentration dependency of K_Na_ modulators and RTG on the depolarization-induced Rb^+^ efflux (see Material and Methods). RTG and BITH enhanced Rb^+^ flux through the recombinant Kv7.2/Kv7.3 currents with EC_50_ of 0.7 ± 0.09 μM and 4.9 ± 1.08 μM, respectively; data were fit to the logistic equation (see Materials and Methods); n = 5. Data for LOX and BEP did not meaningfully fit to the logistic equation. BITH, bithionol; RTG, retigabine; LOX, loxapine; BEP, bepridil.

Modulation of K_Na_ and K_ATP_ channels had more modest effects on E_rest_. K_Na_ blocker bepridil (BEP, 20 μM) [127] depolarized 11/20 (55%) neurons by 5.9 ± 0.7 mV. A recently identified K_Na_ enhancer loxapine (LOX; 10 μM) [9] produced only modest hyperpolarization of −1.5 ± 0.3 mV in a proportion of neurons (4/9; 44%; see Figs. 1A, C and 2C). Another K_Na_ enhancer, bithionol (BITH, 10 μM) [126], [127], produced stronger hyperpolarization of −10.2 ± 0.7 mV in 14/19 (83%) neurons. However, we found that BITH also potently enhanced recombinant M channels (Kv7.2/Kv7.3) with EC_50_ of 4.9 ± 1.08 μM (Fig. 3A, B). Therefore, BITH has been excluded from further analyses. BEP and LOX had no effect on recombinant M channel activity (Fig. 3A, B). The K_ATP_ enhancer pinacidil (PIN; 10 μM) induced a moderate hyperpolarization of −4.6 ± 0.3 mV in 8/11 (73%) neurons (Figs. 1 and 2F), which was significantly weaker than that produced by RTG (Table 3). In contrast, the K_ATP_ blocker glibenclamide (GLIB; 10 μM) failed to produce an effect. This suggests that functional K_ATP_ channels are present in many nociceptors, in accordance with recent discovery of analgesic efficacy of peripheral injections of PIN [27], but they are not active at rest and do not contribute significantly to E_rest_.

**Fig. 4:**
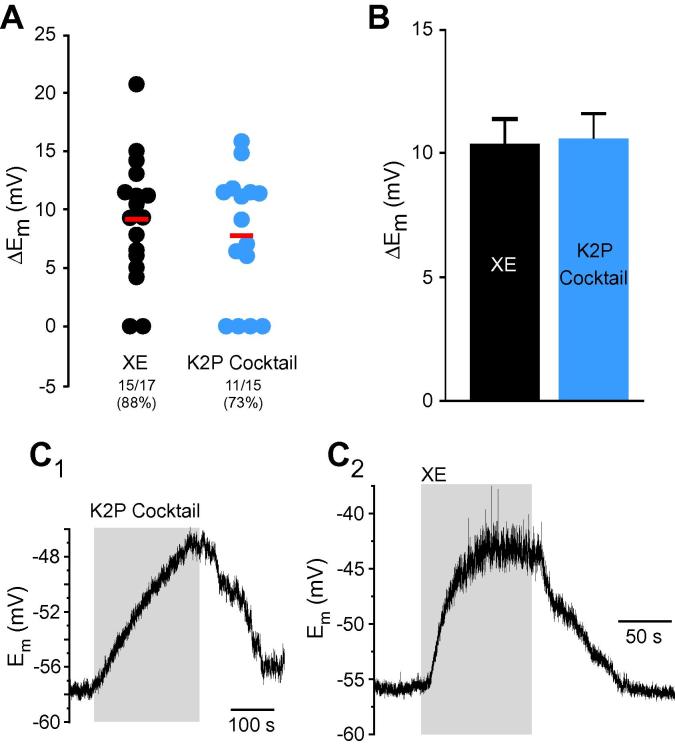
Effects of two-pore K^+^ “leak” channel (K2P) and M channel inhibition on the resting membrane potential of small dorsal root ganglia neurons at 37°C. (A) Scatter plot similar to that shown in Fig. 1B, but the recordings were made at 37°C. (B) Bar chart summarizes effects of XE991 (XE; 3 μM) and a cocktail of K2P inhibitors (100 μM BUP, 100 μM LAM, and 100 μM DOX) taking into account responsive neurons only. (C_1_, C_2_) Exemplary experiments are depicted. BUP, bupivacaine; LAM, lamotrigine; DOX, doxapram.

Several K2P channel subunits (ie, TASK1-3, TREK1-2, TRAAK and TWIK-1-2, and TRESK) are expressed in DRG [80], [115] with TREK-1, TREK-2, and TRESK abundant in small-diameter nociceptors ([1], [53]; reviewed in [26]). Unfortunately, all available pharmacological modulators of K2P channels are not very selective, therefore we tested the effect of 4 different K2P channel blockers on DRG neuron’s E_rest_: bupivacaine (BUP, 100 μM; inhibits TREK-1, TASK-1, and TASK-2, maybe others [83]), lamotrigine (LAM, 100 μM; inhibits TRESK in DRG [54]), doxapram (DOX, 10 μM; inhibits TASK-1, TASK3 [83]), and hydroxyl-α-sanshool (SAN, 100 μM; inhibits TASK-1, TASK-3, and TRESK [8]). All 4 blockers produced a very similar effect, each impacting about 50% of neurons (Figs. 1 and 2G, I); BUP, LAM, DOX, and SAN depolarized 5/9 (56%), 5/10 (50%), 10/17 (59%), and 4/10 (40%) neurons by 6.5 ± 1.4 mV, 4.8 ± 0.9 mV, 4.9 ± 0.4 mV, and 6.3 ± 1.4 mV, respectively. We also tested the K2P channel enhancer, riluzole (RIL; 100 μM [83]), which hyperpolarized 11/19 (58%) DRG neurons by −5.7 ± 0.8 mV (Figs. 1 and 2H); the effect was significantly less strong as compared to RTG (Table 3). RIL also inhibits persistent Na^+^ current [120], thus the effect of K2P enhancement on E_rest_ under our recording conditions can be an overestimation. The effect of K2P modulators was somewhat underwhelming given the previous estimates; thus, Kang and Kim [53] reported that TREK-2-like channels alone contributed ∼70% of the resting K^+^ current in a third of small-diameter cultured rat DRG neurons. siRNA TRECK-2 knockdown resulted in a more depolarized E_rest_ (by about 10 mV) in a sub-population of isolectin B4 (IB4)-positive small-diameter nociceptors [1]. Since TREK-2 and TRAAK display high sensitivity to temperature and are much more active at 37°C compared to room temperature [52], we tested if contribution of K2P channels to E_rest_ is higher at 37°C. In these experiments we used a cocktail of K2P inhibitors (100 μM BUP, 100 μM LAM, and 100 μM DOX) to evaluate the effect of the entire pool of K2P channels. Indeed, we found that at 37°C, the cocktail of K2P inhibitors depolarized E_rest_ by 10.6 ± 1.0 mV in 11/15 (73%) small DRG neurons (Fig. 4), which is in good agreement with [1]; the effect was significantly stronger as compared to the effects of LAM and DOX at room temperature (Mann-Whitney test with Bonferroni correction). Inhibition of M channels with XE at 37°C produced very similar effect: depolarization by 10.4 ± 1.1 mV in 15/17 small DRG neurons (88%). XE effect at 37°C had a tendency to be slightly higher and more prevalent as compared to that at room temperature, but this did not reach significance (Mann-Whitney test, Fisher’s exact test). These experiments suggest that at 37°C, the contribution of K2P channels to E_rest_ in the studied population of small DRG neurons is higher and is similar to that of M channels.

We then turned our attention to channels that generally produce inward currents. Hyperpolarization-activated nonselective cation channels HCN underlie neuronal I_h_; HCN1 and HCN2 are expressed in small-diameter nociceptors [30], [31]. The HCN blocker ZD7288 (ZD; 10 μM) produced moderate hyperpolarization by −3.5 ± 0.5 mV in 11/19 (58%) neurons, suggesting that there is detectable background HCN activity in many cultured DRG somata (Figs. 1 and 2B). The effect of ZD was significantly less pronounced as compared to RTG (Table 3). The effect of ZD was not increased when measurements were repeated at 37°C (not shown).

**Fig. 5:**
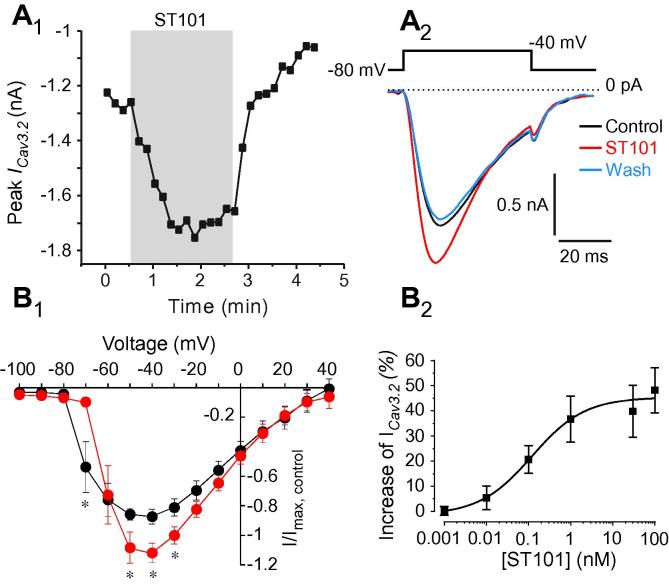
ST101 is a Ca_v_3.2 channel enhancer. (A_1_, A_2_,) Effects of ST101 (1 nM) on the recombinant Ca_v_3.2 channels heterologously overexpressed in HEK293 cells. Recordings were made with whole-cell patch clamp; voltage protocol is depicted above the traces in (A). Shown in (A) is a time-course of the effect of ST101 application (indicated by the grey bar) on the peak Ca_v_3.2 current amplitude. Shown in (A) are the exemplary current traces; dotted line represents 0 pA current level. (B_1_) Mean current-voltage relationships for the recombinant Ca_v_3.2 before (black circles) and after (red circles) the application of 1 nM ST101 (n = 7). ∗ Denotes difference from baseline at *P* ⩽ 0.05 (paired *t*-test). (B_2_) Concentration dependency of the ST101 augmentation of Ca_v_3.2; EC_50_ = 0.12 nM ± 0.04 nM; maximal increase of peak current amplitude = 45.3 ± 3.2%; n = 3-9 for individual data points; the data were fit to the logistic equation (see Materials and Methods).

Most voltage-gated Ca^2+^ channels activate at voltages more depolarized than −60 mV, with the exception of T-type Ca^2+^ channels (Ca_v_3), which have an activation threshold near or even below this voltage [94]; Ca3.2 is the predominant subunit in small-diameter nociceptors [102], [114]. The T-type Ca^2+^ channel blocker mibefradil (MIB; 3 μM) did not significantly affect E_rest_; it only produced a modest 1-mV hyperpolarization in 1 of 6 (17%) neurons, suggesting that T-type channels in most nociceptors at rest are not active. T-type Ca^2+^ channels are also potently and selectively inhibited by the carbon monoxide donor CORM-2 [11]. However, similar to MIB, CORM-2 (3 μM) produced only marginal 1-2 mV hyperpolarization in cultured DRG neurons (not shown). It has been reported that spiro[imidazo[1,2-a]pyridine-3,2-indan]-2(3H)-one (ST101; ZSET1446) potentiates Ca_v_3.1 currents with sub-nanomolar potency [88]. Therefore, we tested the effect of ST101 on E_rest_ in DRG. However, since DRG express little (if any) Ca_v_3.1, and the main T-type Ca^2+^ channel subunit in these neurons is Ca_v_3.2 [114], we first tested the effect of ST101 on the recombinant Ca_v_3.2 channels. Indeed, currents through human Ca_v_3.2 overexpressed in CHO cells were potently augmented by ST101 in a concentration-dependent manner (Fig. 5A-B; EC_50_ = 0.12 ± 0.04 nM, maximal effect at 45.4 ± 3.2%). When applied to DRG neurons, 1 nM ST101 induced depolarization of 3.8 ± 1.2 mV in 9/14 (64%) small neurons tested (Figs. 1 and 2L). The fact that ST101 did not affect Ca_v_3.2 voltage dependence significantly (Fig. 5B_1_), but strongly increased current amplitudes at voltages between −60 and −30 mV, suggests that at E_rest_ of ∼−60 mV native T-type Ca^2+^ channels in DRG neurons are just on the margin of their activation threshold, and thus, may be activated by relatively small depolarizations.

Finally, we tested the contribution of VGNC; small, nociceptive DRG neurons most abundantly express tetrodotoxin (TTX)-sensitive Na_v_1.7, Na_v_1.6, and TTX-resistant Na_v_1.8 and Na_v_1.9 VGNC subunits; reviewed in [21]. In a small fraction of neurons (4/43, 9%), the blocker of TTX-resistant VGNC Na_v_1.8 and Na_v_1.9, A803467 (A803; 10 μM) induced sizable hyperpolarization of −10.3 ± 0.5 mV (Figs. 1 and 2J). These few neurons that did respond to A803 had a tendency to be more depolarized as compared to an average small DRG neuron (−46.2 ± 4.6 mV, n = 4 vs. −61.4 ± 3.1 mV, n = 43). It is possible that heterogeneous expression of these channels contributes to a more depolarized E_rest_ in these particular neurons. TTX-sensitive VGNC may also contribute to E_rest_ [24]. Accordingly, in most neurons (9/10; 90%), TTX induced a small hyperpolarization of −3.3 ± 0.3 mV (Figs. 1 and 2K). Effects of both A803 and TTX were significantly less strong as compared to RTG (Table 3).

### 3.2 Acute DRG slices

Dissociated DRG neurons in culture provide for a convenient experimental model. However, these cells are axotomized and maintained in vitro, and thus, may not necessarily maintain their native phenotype; therefore, data obtained from such neurons may not reflect the physiology of a neuron in situ. We therefore performed additional experiments on neurons in acutely prepared DRG slices. As can be seen from Table 2, the basic electrophysiological characteristics of DRG somata as recorded with sharp intracellular electrodes are quite similar to these recorded with whole-cell patch electrodes (somewhat higher rheobase seen in sharp electrode recordings is likely to reflect larger leak currents introduced by sharp microelectrodes). Particularly relevant to this study is the fact that the values of E_rest_ of cultured and acute DRG neuron somata are within the same voltage range; this ensures that the ion channels that remain active near −60 mV (which we have focused on in this study) are likely to be relevant to the maintenance of E_rest_ of DRG somata both in situ and in vivo.

**Fig. 6:**
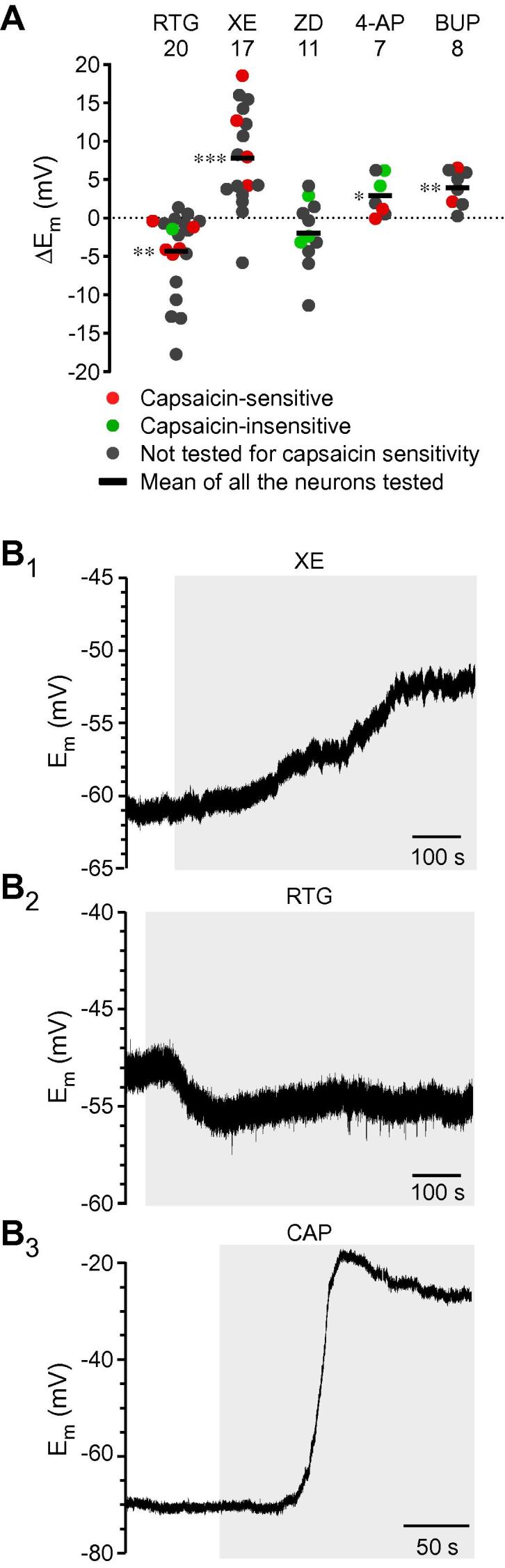
Effect of ion channel modulators on the resting membrane potential of dorsal root ganglia (DRG) neurons recorded with sharp microelectrodes from the acute DRG slices. (A) Scatter plot of changes of membrane potential (ΔE_m_) in DRG slice recordings in response to the application of ion channel modulators. Values from capsaicin-sensitive (red) and -insensitive (green) neurons, as well as from neurons untested for capsaicin sensitivity (dark grey) are identified. Horizontal black bars in every group depict mean values of all neurons tested. Number of recordings is indicated above each group. ^∗^, ^∗∗^, and ^∗∗∗^ denote difference from baseline at *P* ⩽ 0.05, *P* ⩽ 0.01, and *P* ⩽ 0.001, respectively (paired Wilcoxon test). (B) Exemplary time courses of E_m_ recorded using sharp-electrode method from fresh DRG slices during application of XE (B_1_), RTG (B_2_), and CAP (B_3_). XE, XE991; RTG, retigabine; CAP, capsaicin.

In acute DRG slices, we confirmed the key results obtained with cultured DRG neurons (Fig. 6). Since the E_rest_ baselines recorded with sharp electrodes from DRG slices were on average noisier as compared with these recorded using the whole-cell current clamp from cultured DRG neurons, it was difficult to unambiguously classify slice recordings as “responding” or “nonresponding” to a compound (with our threshold parameter for response being set at ΔE_m_ ⩾ 1 mV). Therefore, presented in Fig. 6A is a scatter plot showing all the individual responses recorded in such experiments; mean ΔE_m_ data recorded from all neurons in each group are also given (horizontal black bars). These mean values are likely to underestimate true effects due to the contribution of nonresponding cells. Again, the strongest effect on E_rest_ was found in response to enhancing or blocking M channels; thus, XE depolarized and RTG hyperpolarized E_rest_ by 7.8 ± 1.2 mV (n = 20) and −4.4 ± 1.2 mV (n = 17), respectively. Blocking of HCN channels with ZD (50 μM) resulted in more modest hyperpolarization (−2.0 ± 1.3 mV; n = 11), while inhibition of K2P channels with BUP and K_V_ channels with 4-AP induced comparably more moderate depolarization: 3.96 ± 0.89 mV; n = 8 and 2.87 ± 1.08 mV, n = 7, respectively).

It also has to be noted that in these sharp electrode recordings we were unable to select neurons by size, and therefore, these recordings were blind from a randomized neuron population. In some neurons we were able to test sensitivity to capsaicin (Fig. 6A), but it was not always possible to apply multiple drugs due to the recording stability issues. However, since the majority (up to 70%) of cell bodies in DRG are small-diameter nociceptors [37], [64], [86], it is reasonable to suggest that the majority of our sharp-electrode recordings were indeed performed on this type of neurons. This is consistent with the observation that among the neurons that were tested for the capsaicin sensitivity in this recording paradigm, 23/38 (60.5%) were capsaicin sensitive. These considerations suggest that our sharp electrode recordings are largely representative of nociceptive neurons.

### 3.3 Effect of somatic/perisomatic hyperpolarization on pain signalling in vivo

**Fig. 7:**
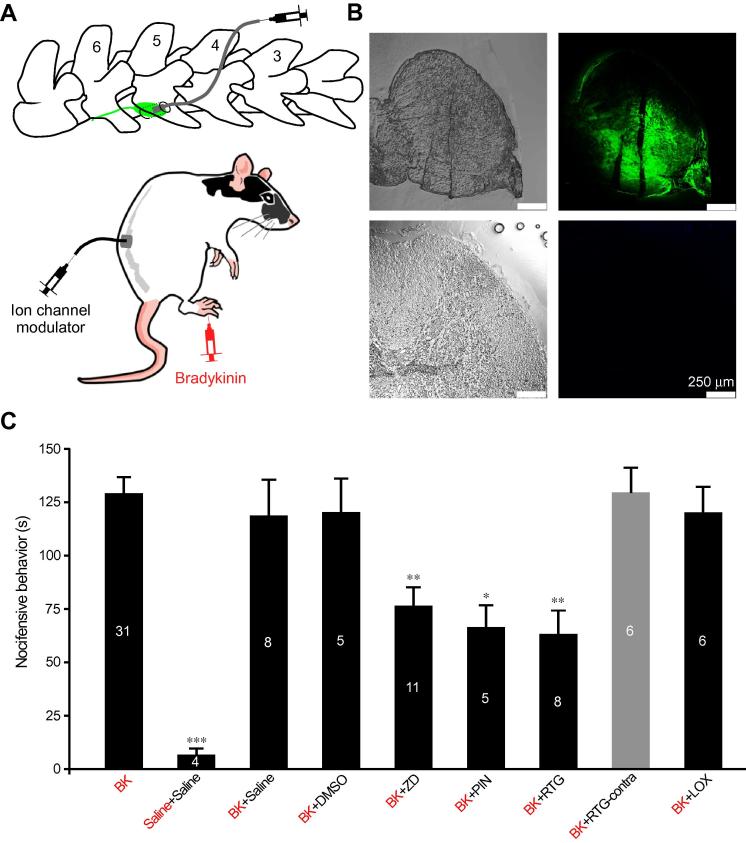
Focal application of ion channel modulators to dorsal root ganglia (DRG) reduces peripheral nociceptive transmission in rats in vivo. (A) Schematic of the DRG cannula implant (modified from [96]). (B) Brightfield (left) and fluorescent (right) images of DRG (top) and proximal dorsal spinal cord (bottom) after the focal application of a fluorescent dye, 5(6)-Carboxyfluorescein diacetate *N*-succinimidyl ester (20 μM in 5 μL) via the DRG cannula (see Materials and Methods). Only the dorsal section of the spinal cord is shown, however, similar lack of staining has been seen in all other sections. (C) Focal ipsilateral application of ZD, PIN, or RTG to DRG reduces nocifensive behaviour produced by hind paw injection of bradykinin (BK; 200 μM, 50 μL). Focal application of LOX (200 μM, 5 μL) and application of RTG to L5 DRG contralateral to the side of BK injection (grey bar) had no effect on BK-induced nocifensive behaviour. Bar chart summarizes the time of nocifensive behaviour (time spent licking, flinching, and biting the paw) over the period of 30 minutes after the BK injection. Immediately prior to the hind paw injection of compounds, animals were given injection of RTG (5 μL, 200 μM), ZD (5 μL, 200 μM), PIN (5 μL, 200 μM), saline (5 μL), or 0.1% dimethyl sulfoxide (DMSO) in saline, 5 μL. From left to right columns correspond to the following injections: hind paw injection of BK only (“*BK*”); hind paw saline + focal DRG application of saline (“*Saline+Saline*”); hind paw BK + focal saline (“*BK+Saline*”); hind paw BK + focal DMSO (“*BK+DMSO*”); hind paw BK + focal ZD (“*BK+ZD*”); hind paw BK + focal PIN (“*BK+PIN*”); hind paw BK + focal RTG (“*BK+RTG*”); hind paw BK + contralateral focal RTG (grey bar; “*BK+RTG-contra*”); hind paw BK + focal LOX (“*BK+LOX*”). In the column labels, red font denotes plantar paw injections and black font denotes focal application to DRG through the cannula. ^∗^, ^∗∗^, and ^∗∗∗^ denote difference from baseline at *P* ⩽ 0.05, *P* ⩽ 0.01, and *P* ⩽ 0.001, respectively (one-way analysis of variance with Bonferroni posttest). ZD, ZD7288; PIN, pinacidil; RTG, retigabine; LOX, loxapine.

In order to evaluate the importance of somatic/perisomatic E_rest_ on nociceptive transmission in vivo, we adapted a method of focal DRG drug injection developed by Puljak and colleagues [96] (with modifications; see Materials and Methods). In this approach, a cannula is inserted into a hole drilled through the transverse process of L5 vertebra; the cannula allows delivery of small volumes of drugs directly to the DRG (Fig. 7A, top panel). In order to verify that this injection technique delivers drugs specifically localized to DRG, and there is no “spill over” to the spinal cord, we injected a fluorescent dye 5(6)-Carboxyfluorescein diacetate *N*-succinimidyl ester (5 μL; 20 μM) through the cannula and tested the extent to which dye spread from the DRG to the proximal spinal cord. Confocal fluorescent imaging revealed abundant fluorescence in the DRG but a complete lack of staining in the spinal cord (Fig. 7B, see Materials and Methods for the experimental details).

Next, we tested the effects of focal DRG application of ion channel modulators on the pain induced by the hind paw injection of bradykinin (BK). BK is a potent endogenous proinflammatory and pain-inducing peptide (algogene) [25], [56]; it produces obvious protective or “nocifensive” behaviour when injected into the hind paw of rats (flinching, biting, and shaking of the injected paw) [76]. Accordingly, injection of BK (50 μL of 200-μM solution) into the hind paw of cannula-implanted rats induced strong nocifensive behaviour, which was not affected by the focal preinjection of vehicles (0.1% dimethyl sulfoxide or saline) to DRG (Fig. 7C; see Materials and Methods for further details).

We then tested compounds that significantly hyperpolarized E_rest_ based on our in vitro experiments. Specifically, drugs that enhanced the activity of M and K_ATP_ channels or blocked HCN channels were injected into the DRG prior to BK administration to the ipsilateral hind paw. Focal preapplication of RTG (5 μL, 200 μM), PIN (5 μL; 200 μM), or ZD (5 μL; 200 μM) significantly attenuated BK-induced nocifensive behaviour (*P* ⩽ 0.01; one-way ANOVA with Bonferroni post hoc test, Fig. 7C). RTG produced the largest attenuation, although difference with PIN and ZD did not reach significance (one-way ANOVA with Bonferroni post hoc test). Importantly, focal DRG application of RTG to the L5 DRG contralateral to the side of BK injection did not produce any reduction in nocifensive behaviour (Fig. 7C; one-way ANOVA with Bonferroni post hoc test). This complete lack of effect of the contralateral focal RTG application is another strong piece of evidence against any spinal effects of a drug applied to DRG via cannula: dorsal roots that ascend from DRG to the spinal cord in rats are about 3 cm long [112], thus, a substance that diffused that distance and reached the spinal cord in a concentration sufficient to produce an effect would definitely produce a bilateral action. K_Na_ enhancer LOX, which only produced marginal hyperpolarization in cultured DRG neurons, was without an effect (Fig. 7C).

It has to be acknowledged that while focal application of drugs to DRG via cannula resulted in no significant spill-over to the spinal cord, the drugs applied in such a manner would affect not only DRG somata but also a perisomatic compartment: stem, t-junction, and adjacent segments of peripheral and central axon. In order to investigate how somatic/perisomatic hyperpolarization may affect transmission of nociceptive signals from the periphery to the spinal cord via the t-junction, we developed a computational model of a small-diameter unmyelinated DRG neuron.

### 3.4 Influence of DRG hyperpolarization on axonal AP propagation: a computational model

**Fig. 8:**
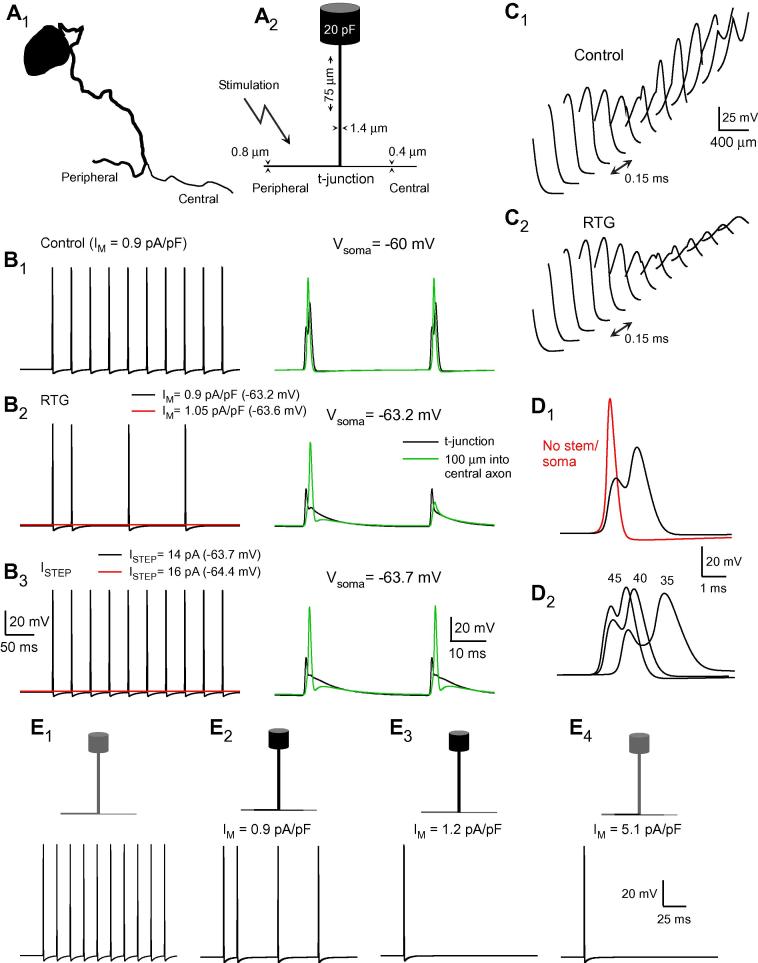
Biophysical model of a small-diameter unmyelinated dorsal root ganglia (DRG) neuron. (A) Morphology of a small-diameter unmyelinated DRG neuron. (A_1_) Drawing of the somatic and perisomatic regions of a small-diameter cat DRG neuron taken from [35] to illustrate the relative geometry of the peripheral, central, and stem axons, as well as the soma. (A_2_) Morphological dimensions of the model neuron. The stem axon (1.4 μm diameter, 75-200 μm length) arising from the soma (25 μm diameter) bifurcates into the unmyelinated peripheral axon (0.8 μm diameter) and central axons (0.4 μm diameter). (B) Enhancing M channels reduced spike propagation in the model neuron. M channels were added to the soma, stem axon, as well as peripheral and central axon segments proximal to the T-junction. They were enhanced in a manner based on the effects of retigabine (RTG); activation was shifted by −30 mV and conductance density was increased 1.5-fold. With an initial M current (I_M_) density of 0.9 pA/pF (at a V_1/2_ = −30 mV; B_1_) spike propagation was reliable. The simulated effect of enhancing M channels with RTG hyperpolarized the soma and reduced spike propagation (B_2_). When the initial I_M_ density was increased to 1.05 pA/pF, RTG enhancement blocked spike propagation. In contrast, somatic current injection that achieved slightly greater hyperpolarization failed to block spike propagation (B_3_). Only when hyperpolarization was at least 4.4 mV below E_rest_ was propagation blocked. (C) M channel activation reduces the likelihood of spike generation in the central axon. (C_1_) Action potential (AP) amplitude is plotted as function of distance along 100 μm of peripheral and central axon surrounding the t-junction. The interval between each trace is 0.15 ms. Under basal conditions, spikes approaching the t-junction decreased in amplitude, consistent with the approaching impedance load of the bifurcation. On the central side of the t-junction, a delayed AP arises and grows with distance from the t-junction. Note that voltage-gated Na^+^ channels (VGNC) density is uniform throughout all 3 axonal regions. (C_2_) Under RTG conditions, the amplitude of the spikes approaching the t-junction was not significantly affected. In contrast, the spike fails on the central side of the bifurcation and the transient decays and broadens with distance from the t-junction. (D) VGNC density and axonal bifurcation are critical determinants for the low-safety factor of spike propagation. (D_1_) Simulation with and without the stem axon connected to the peripheral and central axons demonstrating the effect of the bifurcation on spike amplitude at the t-junction. (D_2_) VGNC conductance (G_Na_) density affects spike waveform at the t-junction. G_Na_ density was varied between 35, 40, and 45 mS/cm^2^. (E) Effect of the spatial distribution M channel modulation. At an initial I_M_ density of 0.9 pA/pF, RTG enhancement of M channels in the soma and stem axon, as well as proximal peripheral and central axon segments, prevented spike propagation (E_2_). However, when M channels were restricted to only the soma and stem axon, spike propagation was reliable, as in (E_1_). Spikes failed when initial I_M_ density was raised to 1.2 pA/pF (E_3_). Likewise, when RTG enhancement was limited to only the axon segments proximal to the t-junction, spikes reliably propagate into the central axon; initial I_M_ density had to be raised to 5.1 pA/pF to limit spike propagation (E_4_).

We have shown that the injection of compounds into the DRG that hyperpolarize sensory neurons in vitro (eg, RTG and ZD) reduced nocifensive behaviour in vivo. Our working hypothesis is that exogenously induced hyperpolarization enhances the likelihood of AP failure across the t-junction, the point of lowest safety factor for AP propagation [19], [33], [79], [109], and in turn reduces the nocifensive response. Successful AP propagation through a branch point depends on the relative impedance loads between the parent axon and its daughters, as well as their active properties, the length of the stem axon, and any contribution by the soma (depending on its electrotonic proximity). Using the dimensions for peripheral, central, and stem axons from the literature ([35], [45], [112]; Fig. 8A), we developed a reduced biophysical model of the DRG portion of an adult, mammalian small-diameter unmyelinated neuron to determine how variations in membrane potential produced by ion channel enhancement or blockade affects AP propagation. Active conductances were limited to a fast VGNC conductance (G_Na_) between the activation ranges of Na_V_1.7 and Na_V_1.8 channels, a delayed rectifier K^+^ conductance (G_KDR_), KCNQ current (M channels), and HCN channels. Channel densities (G_Na_ and G_KDR_) were adjusted to achieve reliable propagation up to a stimulus frequency of approximately 100 Hz [33]. The conduction velocity of the model neuron was 0.33 m/s, as expected for thin, unmyelinated fibres [33], [62]. Somatic APs were 76 mV, somewhat higher than in our recordings of 60-70 mV, but within values reported in the literature [33], [79], [129]. The goal of the model was to identify those factors limiting AP propagation from the periphery to the spinal cord, rather than to build a highly detailed and complete reconstitution of nociceptive neuron electrical response.

Simulated 30-Hz trains of APs initiated in the peripheral axon, a firing frequency within the upper range for unmyelinated nociceptive fibres [14], [108], reliably propagated through the DRG (Fig. 8B_1_). Potentials at the t-junction had a characteristic waveform; the spike invading the bifurcation was reduced in amplitude and was immediately followed by a larger spike generated in the stem axon. As the AP progressed into the central axon, its amplitude increased with distance from the t-junction. At this stimulus frequency, spike propagation was reliable with M channel current (I_M_) densities from 0 and up to 240 pA/pF, while densities of 2.5 to over 20 pA/pF are reported in the literature [17], [74], [93], [100].

We then simulated the effects of enhancing M channels with RTG by increasing I_M_ 1.5-fold and shifting V_1/2_ to −60 mV [72], [118]. The minimal initial I_M_ density required to produce a failure of spike propagation through the DRG, determined as the loss of at least one action potential during a 30-Hz train of 10 spikes, was 0.9 pA/pF (Fig. 8B_2_), which is on the lower end of what was reported in the literature [17], [74], [93], [100]. Under RTG conditions (I_M_ density = 1.35 pA/pF and V_1/2_ = −60 mV), somatic membrane was hyperpolarized by −3.2 mV. All APs reaching the t-junction were reduced in amplitude, but only 4 of the 10 spikes triggered a regenerative and propagating AP in the central axon. As reported previously, AP generation at the soma was not required for AP propagation from the peripheral axon to the central axon [3]. When I_M_ density was increased to 1.575 pA/pF (initial I_M_ density  = 1.05 pA/pF) and V_1/2_ was set to −60 mV, somatic E_m_ hyperpolarized to −63.6 mV and AP propagation through the DRG was completely abolished, not only for high-frequency stimulation, but for lower frequencies (<30 Hz) as well (Fig. 8B_2_). The transition between reliable conduction and failure occurred through a small voltage window, with the hyperpolarization produced by M channel enhancement.

Was AP failure due to hyperpolarization, the increased membrane conductance, or both? To answer this question we tested whether comparable hyperpolarization produced by somatic current injection interfered with AP propagation. Hyperpolarizing the soma by −3.7 mV with constant somatic current (−14 pA) had no effect on the reliability of AP propagation (Fig. 8B_3_). Only when somatic hyperpolarization was further increased to −64.4 mV (−4.4 mV below E_rest_) was spike propagation blocked (Fig. 8B_3_). These results suggest that enhancing M channels reduced AP transmission across the model’s t-junction by both hyperpolarization and increased membrane conductance and, moreover, support the hypothesis that the physiological effects of RTG observed in vitro (see Fig. 6) are sufficient to account for its effects on AP propagation in vivo.

To further characterize how RTG conditions affect spike propagation across the t-junction, in the model we plotted E_m_ as a function of distance along the peripheral and central axonal segments flanking the t-junction at regular time intervals (0.15 ms) under basal and RTG conditions. Under basal conditions (I_M_ density = 0.9 pA/pF, V_1/2_ = −30 mV), as the AP invaded the t-junction, its amplitude decreased to a minimum at the t-junction (Fig. 8C_1_). A regenerative spike in the central axon was subsequently generated and increased in amplitude with distance from the t-junction. In contrast, under RTG conditions (Fig. 8C_2_), the spike leaving the t-junction decreased in amplitude and widened with distance in a manner more consistent with the passive spread of potential.

As stated above, the low safety factor for AP propagation at the t-junction depends on the combined contributions of axonal bifurcation and the active properties of the membrane. The importance of the bifurcation is illustrated in Figure 8D_1_, where a spike invading the t-junction segment is plotted with and without a connected stem axon and soma. The presence of bifurcation results in a significant reduction in spike amplitude localized to the t-junction. The second later peak, arising from the regenerative spike in the stem axon, was also reduced. As a result, enhancing M channels in the central axon alone, distal from the t-junction, failed to block AP transmission under retigabine conditions using I_M_ density values of 45 pA/pF and V_1/2_ = −60 mV (not shown). Membrane excitability at the t-junction and surrounding axons also influenced the amplitude of the invading spike. For example, varying G_Na_ density affected the spike waveform at the t-junction (Fig. 8D_2_) and, in turn, the reliability of AP propagation; in general, raising G_Na_ density elevated the safety factor for spike propagation across the t-junction. Na^+^ channel inactivation had little effect on spike propagation. At the resting potential, 17% of the conductance was inactivated. With hyperpolarization, reactivation of the channels and an increased effective G_Na_ was not sufficient to counter the effects of the lower impedance at the t-junction and the effect of hyperpolarization.

Stem axon length and diameter strongly affected the impedance relationship between parent and daughter axons, and as a result, affected AP propagation in the model. With longer stem axons of 200 μm or greater, basal I_M_ density had to be increased to 13.5 pA/pF (V_1/2_ = −60 mV) in order for M channel enhancement to interfere with AP transmission. When the stem axon had an equivalent diameter to the peripheral axon (0.8 μm), thereby decreasing the conductance ratio [(diam_daughter1_)^3/2^ + (diam_daughter2_)^3/2^]/(diam_parent_)^3/2^ [19] from 2.67 to 1.35, the minimal I_M_ density needed to block spikes was 78.3 pA/pF (V_1/2_ = −60 mV).

M channel subunit Kv7.2 expression has been observed not only in the soma, but also in the stem axon and peripheral fibres [100]. Thus, we examined how the spatial distribution of RTG-enhanced M channels at the soma, stem, and flanking axons affected spike propagation. Starting with an initial I_M_ density of 0.9 pA/pF, as above, RTG modulation of M channels only in the soma and stem axon had no effect on spike propagation. Only when the initial I_M_ density was increased to 1.2 pA/pF did RTG enhancement affect spike propagation (Fig. 8E_1-3_). Likewise, RTG enhancement of M channels limited to only the peripheral and central axons flanking the t-junction had no effect on spike propagation until the initial density was increased to 5.1 pA/pF (Fig. 8E_4_), consistent with the differences in surface area. It is important to note that the range of M channel initial densities used in these simulations (∼1 to ∼5 pA/pF) is comparable to, if not less than, the ranges reported in the literature (2.5 to over 20 pA/pF [17], [74], [93], [100]). If M channel densities were indeed higher, RTG enhancement would be even more potent. Lastly, the densities of M channels that blocked propagation were highly dependent on R_N_ and G_Na_; with higher R_N_, fewer M channels were required to achieve comparable hyperpolarization (and vice versa). Likewise, with greater excitability (ie, higher G_Na_), larger M channel densities were needed to block spike propagation (not shown).

**Fig. 9:**
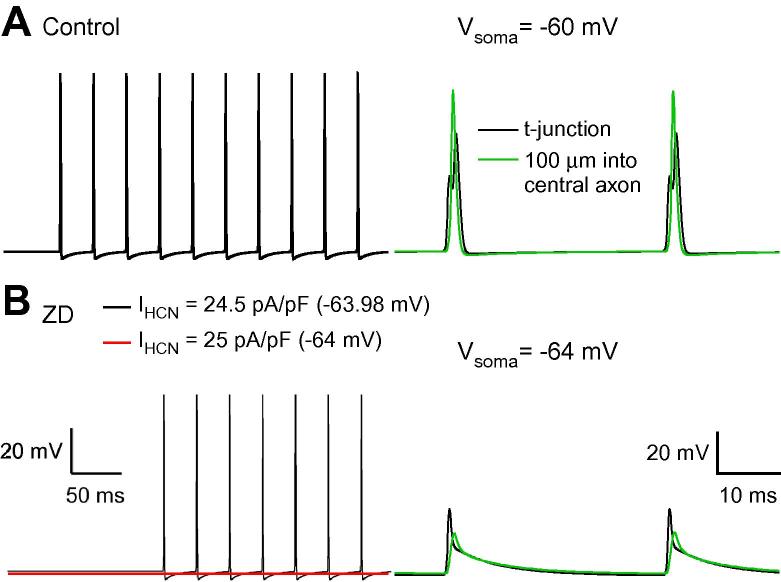
Hyperpolarization produced by blocking hyperpolarization-activated cyclic nucleotide-gated (HCN) channels prevents spike propagation in the dorsal root ganglia model neuron. (A) Starting with an initial HCN channel density of 24.5 pA/pF, spike propagation was reliable in control simulations. (B) Blocking HCN channels (in the soma, stem, and axons proximal to the t-junction), hyperpolarized somatic E_m_, and limited spike propagation. Increasing the initial density to 25 pA/pF resulted in slightly greater hyperpolarization and complete block of spike propagation.

HCN channels are active at the E_rest_ of DRG neurons in vitro, and pharmacological blockade of these conductances hyperpolarizes the membrane potential. Moreover, when HCN blocker is focally applied to the DRG via cannula, it also reduces the nociceptive response in vivo. Blocking HCN channels in our model DRG neuron also hyperpolarized membrane potential, as expected (Fig. 9A). However, hyperpolarization of −3.98 mV below E_rest_ failed to affect spike propagation. In comparison, hyperpolarization of −3.6 mV produced by RTG enhancement of M channels completely blocked AP signalling. Only when membrane potential was hyperpolarized by at least 4 mV was there interference with AP propagation (Fig. 9B). As with the effect of M channel enhancement, the transition between reliable conduction and failure took place within a small voltage range of hyperpolarization produced by HCN channel blockade.

Blocking HCN channels required a greater magnitude of hyperpolarization than observed with M channel enhancement because it not only hyperpolarizes the t-junction (reducing the safety factor for spike propagation), but also reduces total membrane conductance, elevating the safety factor (but to a lesser degree). The effect on spike waveform at the t-junction and the proximal central axon was comparable to that observed for both M channel enhancement and somatic hyperpolarizing current injection. The model predicts that enhancing M channels might be a more potent inhibitor of nociceptive responses than blocking HCN channels; the possible explanation for this discrepancy is discussed below.

## 4 Discussion

Peripheral nociceptive transmission is generally conceptualised as an uninterrupted conduction of peripherally generated APs from their respective sites of origin (eg, skin terminals) to the spinal cord along sensory nerve axons. It has long been recognised that sensory neuron somata are electrically excitable [3], [12], [46], [47], [48], [77], [111], [122], but the cell body and stem axon residing in the sensory ganglia are generally not considered to be important for conduction (eg, [4]). In this study we addressed the following questions regarding somatic excitability of nociceptors and its role in the peripheral nociceptive transmission. 1) what major families of ion channels contribute to the somatic E_rest_? 2) How does manipulating somatic/perisomatic E_rest_ impact transmission of peripherally born nociceptive signals in vivo and in silico?

Firstly, we screened for classes of ion channels that influence E_rest_ of nociceptors. We focused on ion channels that are 1) expressed in small-diameter nociceptive neurons and 2) active at or near −60 mV (ie, M channels, 4-AP sensitive K_V_, K2P “leak,” HCN, K_ATP_, low-threshold Ca_V_, and Na_V_ channels). We found that manipulation of all these channels affected E_rest_ in varying proportions of small-diameter DRG neurons. However, different channels had different efficacies and prevalence within the neuronal population tested. Comparison of effects presented in Fig. 1, Fig. 2 and Table 3 shows the following ranked sequence for hyperpolarizing manipulations: ↑M channels ≫ ↓TTX-sensitive VGNC = ↑K_ATP_ ⩾ ↑K2P ⩾ ↓HCN > ↑K_Na_ > ↓TTX-resistant VGNC ⩾ ↓Ca_v_3. For compounds producing depolarization, the effects were less graded, with inhibition of M channels, K2P, and 4-AP-sensitive Kv channels having stronger and more prevalent effects. Our current clamp recordings were designed to mimic the effects on the E_rest_ of the acute modulation of ion channel activity by endogenous regulatory molecules (eg, inflammatory mediators, cytokines, hormones) released, for example, during acute inflammation or cancer, and also by peripherally active analgesics (for review, see [26], [73], [85]). Therefore, our screen has identified the complement of ion channels that would have the largest effect over the nociceptor’s somatic E_rest_ during such conditions.

M channels were found to have strong influence over E_rest_, as both inhibition and enhancement of M channels caused ∼10 mV de- or hyperpolarization, respectively. M channels are physiologically inhibited by G_q/11_-coupled protein-coupled receptors (GPCR) such as M_1_ mAChR, bradykinin B_2_, protease activated receptor-2, and angiotensin II AT_1_, and such inhibition results in depolarization and increased firing (reviewed in [26], [40], [105]). In contrast, some G_i/o_-coupled GPCR, such as somatostatin receptors, increase M current [87], [98]. In nociceptors, M channels can be augmented by neurokinin receptors [69], [71], an action that results in reduced excitability [71]. Thus, M channels may represent a major endogenous mechanism for tuning the excitability of nociceptive neurons. 4-AP-sensitive Kvs, for example, Kv1.4, Kv2s, and Kv3.4, expressed in small DRG neurons [26] as well as K2P channels, also strongly influenced E_rest_; the K2P channel contribution was significantly higher at 37°C (suggesting large contribution of temperature-sensitive TREK-2 channel, which has low activity at room temperature [52] and is highly expressed in IB4-positive nociceptors [1]). K_ATP_ channels, while present in ∼70% of nociceptors (as evidenced by the hyperpolarization induced by K_ATP_ enhancer, PIN), were not active at rest, as the K_ATP_ inhibitor GLIB failed to depolarize the E_rest_. In contrast, K_Na_ channel inhibition produced moderate depolarization (∼5 mV) in 55% of the neurons; the K_Na_ enhancer LOX produced only a marginal effect.

Among depolarizing currents, we tested the contribution of HCN channels, T-type Ca^2+^ currents, and VGNC. In ∼60% of the neurons, a modest hyperpolarization (relative to that produced by RTG) was observed with the blockade of HCN channels. Inhibition of TTX-resistant Na^+^ channels produced large (∼−10 mV) hyperpolarization of the membrane, but in a very small proportion (9%) of neurons. In the majority of DRG cells, TTX hyperpolarized E_rest_, but only by −2 to −3 mV. Other cationic or anionic background conductances that may potentially also contribute to E_rest_ (eg, persistent currents through ϒ-aminobutyric acid [GABA]_A_ or *N*-methyl-D-aspartate receptors expressed in DRG [66], [67], [124]) were not addressed in the current study.

Our next question was to establish how modulation of “resting” conductances of the somatic and perisomatic compartments of nociceptors affects transmission of sensory information from periphery to the spinal cord. We tested whether compounds that hyperpolarize E_m_ of small DRG neurons interfere with the relaying of APs from the periphery in vivo. Indeed, focal application of 2 K^+^ channel enhancers, RTG and PIN, as well as the I_h_ blocker ZD strikingly attenuated nocifensive behaviour induced by the hind paw injection of BK (Fig. 7). The K_NA_ enhancer LOX, which only produced nominal hyperpolarization in vitro, was without effect.

In order to better understand how these compounds were limiting pain information from reaching the spinal cord, we constructed a computational model of a small-diameter unmyelinated DRG neuron and reached the following conclusions:

1) The morphology of the DRG axon bifurcation based on anatomical measurements [35], [45], [112] fosters an intrinsically low safety factor for AP propagation that has been observed experimentally in amphibian and embryonic DRG neurons [79], [109], as well as adult mammalian C-fibre neurons [33]. An electronically short stem axon, achieved by a short length and/or larger diameter, was essential to the low safety factor. The diameter provides for a larger conductance ratio and both the length and diameter allow potential at the soma to affect potential at the t-junction. Hyperpolarization, produced by enhancing M channels, blocking HCN channels, or somatic current injection, further lowered the safety factor and, as a result, interfered with AP propagation. Increased membrane conductance (induced by somatic/perisomatic K^+^ channel enhancement) additionally contributed to lowering the safety factor. Without a t-junction, the safety factor in the axon is relatively high, and comparable ion channel modulation, for example, in the central axon distal to the t-junction, had no effect on spike transmission. Accordingly, injection of M channel enhancer flupirtine (close analogue of RTG) into sciatic nerve of control rats did not affect nociceptive transmission from the periphery (while similar injection of lidocaine expectedly did) [100]. Recent evidence suggests that the site of analgesic activity of systemically administered RTG is almost exclusively peripheral because, in contrast to its anticonvulsant activity, it was not antagonized by central application of XE [39]. Thus, since the t-junction most likely has the lowest safety factor for AP propagation within the peripheral nociceptive pathway, it is logical to hypothesize that AP failure at nociceptive neuron t-junctions may contribute to the analgesic effect of systemic RTG.
2) The soma of a small-diameter unmyelinated DRG neuron is electrotonically close enough to the t-junction to influence AP transmission. Although excitability (in this case, electrogenesis) at the soma does not normally affect spike propagation [4], hyperpolarization of the soma substantially influences membrane potential at the t-junction. As a result, manipulations that sufficiently hyperpolarize the t-junction interfere with the transmission of APs from the periphery to the spinal cord, again assuming a low safety factor (discussed above).
3) The combination of hyperpolarization and increased membrane conductance should be more potent at blocking spike propagation than either alone. Thus, the efficacy of ZD to attenuate BK-induced pain observed in vivo is not entirely consistent with modelling results. It has been reported recently that I_h_ density was very low in C-fibre nociceptors, but much higher in nociceptive sub-populations of Aδ and Aβ fibres (5, 13, and 21 pA/pF at −100 mV, respectively) [31]. Notably, in our simulations (Fig. 9), HCN channel block interfered with AP propagation only at high HCN channel densities (∼25 pA/pF), suggesting a possible explanation for the weak effect of ZD on E_rest_ in small nociceptors in vitro and relatively strong effect in reducing peripherally induced pain in vivo: the in vivo effect of ZD is likely to be mediated by Aδ and Aβ nociceptors. The model demonstrates proof-of-principle that interference with AP propagation, even at low stimulus frequencies, could be achieved by manipulating somatic/perisomatic conductances in DRG [44], [75], [78].

Importantly, both our in vivo and in silico experiments strongly suggest that somatic/perisomatic compartment of nociceptive neurons has indeed a strong filtering role and may impede incoming APs. Potentially in support of this finding is the recent clinical discovery that electrical stimulation (neuromodulation) of DRG in humans via the implanted electrodes provides efficacious pain relief in patients with various “untreatable” neuropathic pain syndromes [20], [68], [95]. The exact action of such DRG stimulation has yet to be discovered. However, the facts that a) DRG stimulation itself does not cause pain, and b) cessation of stimulation allows pain to return [68], suggests that this type of analgesia arises from the induced failure of peripherally generated APs to pass through the ganglion.

To our knowledge, the experiments presented here are the first to demonstrate that somatic/perisomatic E_m_ can regulate sensory transmission from the periphery to the spinal cord. Thus, the DRG may play a much stronger role in controlling peripheral transmission than generally accepted, representing a hitherto underappreciated additional “gate” within the peripheral nociceptive system.

## Conflicts of interest

The authors declare that they have no conflicting interests.
